# REST deficiency and neurogenic-to-gliogenic shift in down syndrome human cerebral organoids

**DOI:** 10.1186/s13041-026-01313-2

**Published:** 2026-05-16

**Authors:** Tan Huang, Chong-Teik Lim, Wei Li, Sharida Fakurazi, John O. Mason, Pike-See Cheah, Yi Li, King-Hwa Ling

**Affiliations:** 1 Department of Neurosurgery, The Second Affiliated Hospital of Zunyi Medical University, Zunyi City, 563000 Guizhou Province China; 2 Department of Biomedical Sciences, Faculty of Medicine and Health Sciences, Universiti Putra Malaysia, 43400 Serdang, Selangor Malaysia; 3 Department of Human Anatomy, Faculty of Medicine and Health Sciences, Universiti Putra Malaysia, Serdang, 43400 Selangor Malaysia; 4 Institute for Neuroscience and Cardiovascular Research, The University of Edinburgh, Hugh Robson Building, 15 George Square, Edinburgh, EH8 9XD UK; 5 Malaysian Research Institute on Ageing (MyAgeing®), Universiti Putra Malaysia, 43400 Serdang, Selangor Malaysia; 6 M. Kandiah Faculty of Medicine and Health Sciences, Universiti Tunku Abdul Rahman, Cheras, Selangor Malaysia; 7 Fakultas Kedokteran, Universitas Pembangunan Nasional “Veteran” Jakarta, Jakarta, Indonesia

**Keywords:** Cerebral organoid, Down syndrome, hiPSC, Neurodevelopment, REST

## Abstract

Down syndrome (DS) features impaired cortical neurogenesis and excess gliogenesis, yet the temporal regulatory events driving this imbalance remain unclear. Here, we combine multi-timepoint transcriptomic analyses from publicly available datasets, network modelling, and machine-learning prioritization, with validation in isogenic human iPSC-derived cerebral organoids, to identify a discrete pathogenic window at 90 days in vitro (DIV 90). Across five developmental stages, REST target genes were preferentially dysregulated in DS organoids. WGCNA revealed a DS-associated module at DIV-90 that strongly overlapped with REST targets, and two orthogonal machine-learning approaches converged on six REST-regulated hub genes—*CSTB*, *MCM3AP*, *PFKL*, *POFUT2*, *PRMT2*, and *RWDD2B*. In trisomic organoids, REST mRNA and nuclear protein were markedly reduced at DIV-90, accompanied by diminished DCX expression and activation of NFIA and STAT3, suggesting a neurogenic-to-gliogenic fate transition. These findings suggest REST dysfunction as a potential temporal regulator of lineage imbalance in DS and highlight REST-linked networks as potential targets for early developmental intervention.

**Supplementary Information:**

The online version contains supplementary material available at 10.1186/s13041-026-01313-2.

## Introduction

Down syndrome (DS) is a primary genetic condition associated with intellectual disability, resulting from trisomy of human chromosome 21 (HSA21). A disruption in the balance between neurogenesis and gliogenesis is believed to contribute to the cognitive impairments observed in DS. Studies have shown decreased excitatory neurons and interneurons [1, 2], along with a significant increase in astrocytes [3, 4] in the cerebral cortex of individuals with DS. Recent studies have demonstrated that three-dimensional (3D) cerebral organoid models derived from human induced pluripotent stem cells (hiPSC) of individuals with DS can mimic essential characteristics of the human brain, including cellular distribution, organisational patterns, physiological structures, electrical activity, and neural networks [5, 6]. Multiple studies have shown that a third copy of chromosome 21 leads to extensive transcriptomic changes, resulting in genome-wide transcriptional dysregulation [7–9]. The molecular mechanisms underlying this phenotype and the critical developmental time points affected in individuals with DS are still poorly understood. Hence, analysing the various developmental stages of organoids can provide clearer insights into the molecular mechanisms driving the dynamic changes and abnormalities in the DS brain transcriptome during early neurodevelopment.

REST, the Repressor Element-1 Silencing Transcription Factor, is a crucial regulator of gene expression, particularly in neuronal cells. REST is a transcriptional repressor bound to the RE1 binding site on neuronal genes, thereby inhibiting their expression [10]. REST has 15,450 known target genes in the human genome [11]. It is involved in a range of cellular processes, including neuronal development, differentiation, axon elongation, vesicle transport, ion channel signalling, and synaptic plasticity [12]. The level of REST in the human DS foetal brain showed a notable reduction compared to diploid controls [13]. The loss of REST activity results in the derepression of target genes in glial cells and neural cells [14, 15]. Further exploration of the mechanisms underlying REST-mediated molecular dysregulation across different neurodevelopmental stages and the critical time points at which the neurogenic-to-gliogenic shift occurs during DS neurodevelopment is necessary.

In this study, publicly available high-throughput sequencing datasets from brain organoids across multiple developmental stages were utilised to investigate the role of REST target genes in the neurological development and pathogenesis of DS. Experimentally, DS hiPSC-derived cerebral organoids exhibited significantly reduced REST expression and a neurogenic-to-gliogenic shift by DIV 90. Additionally, comprehensive in silico machine learning approaches identified six REST-targeted hub genes associated with DS neuropathology. These genes were upregulated in DS cerebral organoids, supporting a role for REST loss in the neuropathological development of DS. These results indicate that REST is a promising target for developing therapies for individuals with DS.

## Materials and methods

### Acquisition of DS brain organoid datasets

Next-generation sequencing datasets for human cerebral organoids derived from human induced pluripotent stem cells were obtained from the Gene Expression Omnibus (GEO; https://www.ncbi.nlm.nih.gov/geo/). Publicly available transcriptomic datasets of human cerebral organoids across multiple developmental stages (DIV 30–160) are listed in Table 1 (Accession IDs: PRJNA721827, GSE124513, GSE208440, and GSE222365).

**Table 1:** Summary of cerebral organoid transcriptomic datasets analysed in this study

Database ID	DIV stage	DS samples (*n*)	Control samples (*n*)	Total samples (*n*)	Isogenic controls	Reference
PRJNA721827	DIV 30	3	3	6	Yes	[2]
GSE124513	DIV 35	8	8	16	Partially	[16]
GSE208440	DIV 56	3	3	6	Yes	[17]
GSE222365	DIV 90	24	24	48	Yes	[18]
GSE222365	DIV 160	5	5	10	Yes	[18]

## Processing of the dataset and identification of differentially expressed genes (DEGs)

The bioinformatic tools on the Galaxy web platform (https://usegalaxy.org) were used to comprehensively analyse the Next-Generation Sequencing (NGS) dataset. Initial processing involved reading and trimming FASTQ files using Trim Galore (Galaxy Version 0.6.10 + galaxy0) and assessing individual sample quality using FASTQC. Subsequently, the transcriptome sequences were aligned using HISAT2 (Galaxy Version 2.2.1 + galaxy1), with the hg38 human reference genome as the annotation file. The htseq-count software (Galaxy Version 2.0.5 + galaxy0) was used to count aligned reads. To explore differential gene expression between DS and control samples, we employed DESeq2 (Galaxy Version 2.11.40.8 + galaxy0). Genes were classified as differentially expressed genes (DEGs) based on a significance threshold of adjusted p-value < 0.05 and |log2 fold change| > 0.5, including both upregulated (log2 FC > 0.5) and downregulated (log2 FC < − 0.5) genes. The analysis results were visualised in RStudio (version 2025.05.0 + 496), where a heatmap and a volcano plot were generated to represent the findings.

## Identification of REST-targeted DEGs in DS cerebral organoids

The REST target genes specific to humans were extracted from the Harmonizome 3.0 Database (https://maayanlab.cloud/Harmonizome/) [19]. Venn diagrams (https://bioinfogp.cnb.csic.es/tools/venny/) were used to show the overlap between REST target genes and DEGs. A hypergeometric probability model was used to assess the statistical significance of overlap between two gene sets, with the human genome as the background (http://nemates.org). The representation factor was calculated as the ratio of the observed number of overlapping genes to the expected number, based on two independent gene sets. The expected overlap was estimated based on the sizes of the two gene sets relative to the total number of genes in the background set. A representation factor greater than 1 indicates more overlap than expected by chance, whereas a value less than 1 indicates less overlap than expected.

## Gene ontology and pathway enrichment analysis

The ‘clusterProfiler’ [20] bioconductor package in R was used to perform enrichment analysis on Gene Ontology (GO) and Kyoto Encyclopedia of Genes and Genomes (KEGG) [21] terms for DEG and REST target DEG. Enrichment significance was established with stringent criteria of p-value < 0.05 and adjusted p < 0.05 for both GO and KEGG terms, respectively. The ‘dplyr’ and ‘ggplot2’ packages were used to visually represent the comprehensive GO and KEGG results.

## Gene set enrichment analysis

Gene Set Enrichment Analysis (GSEA) [22] was employed to assess enrichment of GO and KEGG pathways, evaluating their statistical significance in DS cerebral organoids compared with the control group. The GSEA analysis and visualisation were performed using the R packages ‘org.Hs.eg.db’, ‘clusterProfiler’, and ‘richplot’. Significance criteria are set at |Normalised Enrichment Score| (NES) > 1, p-value < 1e − 10, and adjusted p-value < 0.05 for robust identification of enriched pathways.

### WGCNA network construction and module identification

Weighted Gene Co-expression Network Analysis (WGCNA) is a systematic biological approach that clusters genes with similar expression patterns, effectively categorising them into modules based on their relevance. In this study, the ‘WGCNA’ [23] package in R was used to construct and visualise the network. Our investigation employed WGCNA on the GSE222365 dataset (DIV 90 organoids). This dataset comprises 24 DS samples and 24 corresponding isogenic control samples. The network development involved using a soft-threshold parameter (β), carefully chosen to achieve a scale-free topological overlap matrix fit value of 0.9. The optimal β value was selected by identifying the lowest power at which the desired scale-free topological overlap matrices fit was attained. Pearson correlation analyses were subsequently conducted to explore the interrelationships among gene modules, specifically identifying the module exhibiting the most robust correlation with DS. Gene Significance (GS) and Module Membership (MM) values were computed. Critical genes associated with the module were identified using stringent criteria, specifically, those with GS > 0.5 and MM > 0.8.

## Machine learning for screening hub genes

Machine learning has emerged as a powerful tool for mining existing gene expression data, enabling the derivation of rules and models to predict pivotal genes in the genome. This study deploys a sophisticated computational method that synergistically incorporates Random Forest (RF) and Least Absolute Shrinkage and Selection Operator (LASSO) logistic regression algorithms. This integrated approach aimed to meticulously sift through genetic data to identify biomarkers specific to the DS brain organoids landscape. Specialised packages, such as ‘glmnet’ for LASSO and ‘randomForest’ for Random Forest, were used to perform this comprehensive analysis. By applying these advanced algorithms, we sought to uncover and characterise significant genetic markers. The convergence of genes identified by the three algorithms is recognised as a set of critical genes, offering insights into the genomic intricacies of DS cerebral organoids.

## In silico validation of hub gene expression

To evaluate the expression patterns of the identified hub genes, gene expression values were extracted from the transcriptomic datasets of DS-derived and control cerebral organoids at different developmental stages. For each dataset, the expression levels of the identified hub genes were compared between DS-derived and control organoids. Statistical comparisons were performed using a two-tailed Student’s t-test. Differences were considered statistically significant when *P* < 0.05. The results were visualised using boxplots to illustrate differences in expression between groups.

### Production of HiPSC-derived cerebral organoids

This study included three trisomic iPSC lines [DS1 (UWWC1- DS1), DS2 (WC-24-02-DS-M), and DS4 (HPS4270)] and three isogenic euploid iPSC lines [(C1 (UWWC1- DS2U), C2 (WC-24-02-DS-A), and C5 (HPS4272)]. DS1, C1, DS2, and C2 were obtained from the WiCell Research Institute at the University of Wisconsin [24, 25]; C5 and DS4 were sourced from the RIKEN BioResource Centre in Japan [26]. Detailed information on the cell lines is provided in Table 2. HiPSCs were cultured in complete mTeSR™ Plus medium (Catalogue # 100–0276; STEMCELL Technologies, Canada) in a six-well plate coated with Geltrex (Catalogue # A1413302; Gibco, USA), adhering to the manufacturer’s guidelines. The STEMdiff™ Cerebral Organoid Kit (Catalogue # 08570, STEMCELL Technologies, Canada) was used for embryoid body (EB) formation, which included EB seeding medium (containing a ROCK inhibitor), EB induction medium, and EB formation medium.

Human-iPSCs were dissociated using Accutase (Catalogue # 07922; STEMCELL Technologies, Canada). Upon reaching approximately 80% confluency, cells were plated onto a 96-well round-bottom ultra-low attachment plate at a density of 9,000 cells per well in 100µL of EB seeding medium. After 2 days, 100µL of EB Formation medium was added to each well. On day 4, the medium was refreshed with an additional 100µL of EB Formation medium. By day 5, the EB Formation medium was removed and replaced with 150 µL of induction medium, and the plates were then incubated at 37 °C for 48 h. For cerebral organoid maturation, the STEMdiff™ Cerebral Organoid Maturation Kit (Cat # 08571, STEMCELL Technologies, Canada) was utilised, which included both expansion and maturation media. On day 7, each organoid was embedded in 30µL of Matrigel and incubated at 37 °C for 30 min. Subsequently, the organoids were transferred to a new six-well ultra-low-adherence plate containing 3 mL of expansion medium and incubated for 3 days. After this incubation, the medium was replaced with 3 mL of maturation medium per well, and the culture was maintained at 37 °C on an orbital shaker at 75 rpm. The medium was refreshed every 3–4 days until the organoids were harvested on DIV 90.

**Table 2:** Details of the disomic control and trisomic DS iPSC lines

Pair ID	Trisomic line (DS)	Euploid control line	Donor origin	Provider
Pair 1	DS1 (UWWC1-DS1)	C1 (UWWC1-DS2U)	• Mosaic DS individual, male • Aged 1 year • Skin fibroblast	WiCell Research Institute
Pair 2	DS2 (WC-24-02-DS-M)	C2 (WC-24-02-DS-A)	• Mosaic DS individual, female • Aged 25 years • Skin fibroblast	WiCell Research Institute
Pair 3	DS4 (HPS4270)	C5 (HPS4272)	• Trisomy 21 individual, male • Aged < 10 years • Skin fibroblast	RIKEN BioResource Centre

C: Control. DS: Down syndrome

### Immunohistochemistry staining

Cerebral organoids were fixed overnight at 4 °C in 4% paraformaldehyde, followed by sequential cryoprotection in 15% and 30% sucrose at 4 °C for 24 h each. Organoids were embedded in optimal cutting temperature (OCT) compound, snap-frozen on dry ice, and stored at − 80 °C. Sections  (20 μm) were prepared using a cryostat (− 20 °C) and mounted on Superfrost Plus slides (Thermo Fisher Scientific). For immunostaining, slides were air-dried at 37 °C for 30 min, rehydrated in PBS, and autofluorescence was quenched using 125 mM glycine. Sections were blocked in 3% normal donkey serum with 0.3% Triton X-100, followed by overnight incubation at 4 °C with primary antibodies diluted in blocking buffer. The next day, slides were washed and incubated for 1 h at room temperature with Alexa Fluor-conjugated secondary antibodies (1:2000). To minimise background, 0.1% Sudan Black in 70% ethanol was applied for 20 min. Nuclei were counterstained with DAPI, and sections were mounted with antifade medium and sealed with nail polish. Primary antibodies included anti-Ki67 (AB9260; Sigma-Aldrich, USA; 1:250 dilution), anti-TBR1 (#49661; Cell Signaling Technology, USA; 1:350 dilution), anti-SOX2 (ab239218, Abcam, USA; 1:250 dilution), anti-REST (22242-1-AP; Proteintech, USA; 1:200 dilution), anti-DCX (CSB-PA006576DA0HU; Cusabio, USA; 1:200 dilution), and anti-NFIA (E-AB-67569; Elabscience, USA; 1:200 dilution). Corresponding secondary antibodies included Alexa Fluor 488 donkey anti-rabbit (A-21206; Invitrogen, USA; 1:2000 dilution) for Ki67, TBR1, REST, DCX, and NFIA; Alexa Fluor 594 donkey anti-goat (ab150132, Abcam, USA; 1:2000 dilution) for SOX2.

### Immunofluorescence image acquisition and quantification

Immunofluorescence images were acquired using a fluorescence microscope under identical exposure settings for all samples. For each cell line and staining condition, at least four independent microscopic fields were captured and analysed. Image analysis was performed using ImageJ software (NIH). Fluorescence images were separated into individual channels, and nuclei were identified from the DAPI channel using threshold-based segmentation. Background fluorescence was estimated from negative control images and subtracted before quantification. For each image, the total fluorescence intensity (Raw Integrated Density) of the marker signal was measured and normalized to the number of DAPI-positive nuclei in the same field to obtain the average fluorescence intensity per cell. All images were analysed using identical analysis parameters to ensure consistency across samples.

### Quantitative real-time PCR

Cerebral organoids were washed and recovered in cold cell recovery solution (354253, Corning, USA) to remove the Matrigel matrix. RNA extraction was performed on the organoid pellets using the Ribospin II extraction kit (#314150, GeneAll, Korea) according to the manufacturer’s protocol. The extracted RNAs were reverse-transcribed into cDNA using LunaScript RT SuperMix kit (E3010, New England Biolabs, UK). RT-qPCR was performed on the cDNA using 2x Luna^®^ Universal qPCR Master Mix according to the manufacturer’s guidelines. The RT-qPCR assays, with a final volume of 20 µl, will be performed using the LightCycler^®^ 480 Real-Time PCR System (Roche Diagnostics, Australia). Samples will undergo duplicate assays, with PSMB2 and HMBS as internal controls. The primers for the RT-qPCR validation are listed in Table 3. The condition for the RT-qPCR induced an initial denaturation step with 1 cycle of 95 °C for 60 s, followed by 45 cycles of 95 °C (denaturation) for 15 s and 60 °C (extension) for 30 s, 1 cycle of 72 °C (melt curve) for 30 s, and finished with a cooling step at 40 °C for 1 min.

**Table 3:** Primer sequences

Primer	Sequence (5’-3’)	Annealing temperature (℃)	Length (nt)
CSTB F	AGGTCCCAGCTTGAAGAGAAA	60	21
CSTB R	CGCAGGTGTACGAAGTCCTC		20
DCX F	GAAGGGAAACCCATCAGCCA	60	20
DCX R	GAGGTTCCGTTTGCGTCTTG		20
MCM3AP F	GGTTGTCTGAAGACTCAGGAGC	60	22
MCM3AP R	TCCCTCATCATGTCACTCTGT		21
HMBS F	GATGTTAGGAGCCCTGTTTGG	60	21
HMBS R	TTACGAGCAGTGATGCCTACC		21
JAK2 F	CCGGGTTTCAGAAGCA	60	16
JAK2 R	TCAGAACATTTGCCGTCG		18
NFIA F	TGAAGTGGAGCCAGGAATGC	60	20
NFIA R	ATGACAGGTCGGTGATGCTG		20
PFKL F	CTACGAGGGCTATGAGGGC	60	19
PFKL R	GATGACGCACAGGTTGGTGA		20
POFUT2 F	GAGCAGTTCATCGCAGAATCT	60	21
POFUT2 R	AACCCCAAAACCATCCTCTGT		21
PRMT2 F	ACCTGCCTGCTGTTTGAGT	60	19
PRMT2 R	CAAATGACCCCGTCCTCCTTC		21
PSMB2 F	GAGGGCAGTGGAACTCCTTAG	60	21
PSMB2 R	GATGTTAGGAGCCCTGTTTGG		21
REST F	GAGAACGCCCATATAAATGTG	60	21
REST R	CACATAACTGCACTGATCAC		20
RWDD2B F	GCCACGGCTCAAGAAACTTAC	60	21
RWDD2B R	TCGCCCCTCCATTGTCTTCT		20
STAT3 F	AGAAGGACATCAGCGGTAAG	60	20
STAT3 R	CGTTGGTGTCACACAGATAA		20

F: Forward Primer, R: Reverse Primer, CSTB: Cystatin B, DCX: Doublecortin, MCM3AP: Minichromosome maintenance complex component 3 associated protein, HMBS: Hydroxymethylbilane synthase, JAK2: Janus kinase 2, NFIA: Nuclear factor I A, PFKL: Phosphofructokinase-liver type, POFUT2: Protein O-fucosyltransferase 2, PRMT2: Protein arginine methyltransferase 2, PSMB2: Proteasome 20 S subunit beta 2, REST: RE1 silencing transcription factor, RWDD2B: RWD domain containing 2B, STAT3: Signal transducer and activator of transcription 3.

F, Forward Primer; R, Reverse Primer; *CSTB*, Cystatin B; *DCX*, Doublecortin; *MCM3AP*, Minichromosome maintenance complex component 3 associated protein; *HMBS*, Hydroxymethylbilane synthase; *JAK2*, Janus kinase 2; *NFIA*, Nuclear factor I A; *PFKL*, Phosphofructokinase-liver type; *POFUT2*, Protein O-fucosyltransferase 2; *PRMT2*, Protein arginine methyltransferase 2; *PSMB2*, Proteasome 20 S subunit beta 2; *REST*, RE1 silencing transcription factor; *RWDD2B*, RWD domain containing 2B; *STAT3*, Signal transducer and activator of transcription 3.

### Statistical analysis

The data are presented as mean ± standard deviation (SD), with all experiments conducted in biological and technical triplicate. Statistical analyses were performed using GraphPad Prism 9.0 (version 9.5.0) or R software (version 2023.03.0 + 386). An independent-samples t-test was used to compare the two independent experimental groups. A significance threshold of *P* < 0.05 was applied, and statistical significance is represented as follows: ns, not significant; *P < 0.05; **P < 0.01; ***P < 0.001; ****P < 0.0001.

## Results

### Differentially expressed genes and their ontologies and pathways in different developmental stages of DS cerebral organoids

DS iPSC-derived organoids from five different time points were screened for DEGs using an adjusted p-value < 0.05 and |log2 FC| > 0.5. In the PRJNA721827 (bulk) dataset (DIV 30), 235 DEGs were identified. In GSE124513 (DIV 35), 3,183 DEGs were identified. In GSE208440 (DIV 56), 6,818 DEGs were identified. In GSE222365 (including DIV 90 and 160 organoids), 923 DEGs were identified in DIV 90 organoids and 3,598 DEGs in DIV 160 organoids. These differential expression patterns are illustrated in the corresponding heatmaps (Figure S1), and the complete list of differentially expressed genes is provided in Supplementary Table S1.

**Fig. 1:**
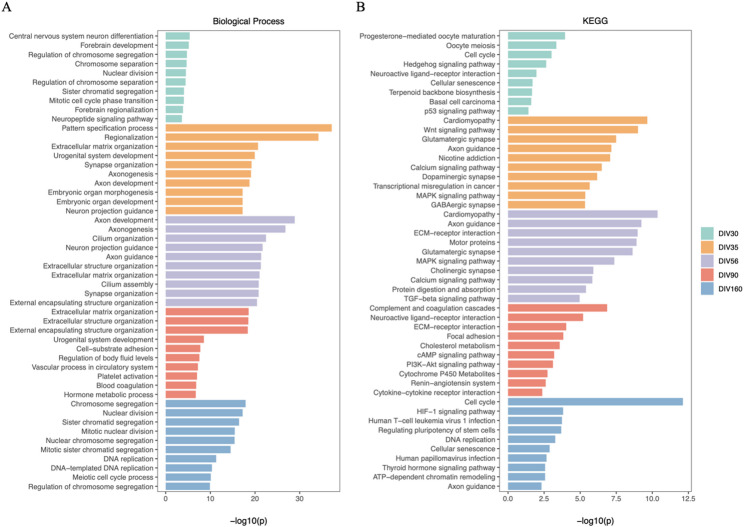
Enrichment analysis of DEGs in DS-derived cerebral organoids at different time points. Different colours indicate different time points in DS cerebral organoids. The top 10 biological processes **A** and KEGG pathways **B** for each time point are shown

To investigate the functional and mechanistic characteristics of DS iPSC-derived organoid development, we performed GO and KEGG enrichment analyses of DEGs at different time points (Fig. 1). The GO biological process results reveal that the DEGs in DIV 30 organoids are mainly enriched in chromosome segregation, mitotic cell cycle, and nuclear division. The KEGG pathway analysis shows that the DEGs in DIV 30 organoids are primarily involved in the cell cycle, oocyte meiosis, and neuroactive ligand-receptor interaction. In DIV 35 organoids, the GO biological processes of the DEGs were mainly enriched in axon development, axonogenesis, synapse organisation, and neuron projection guidance. The KEGG pathway predominantly highlights involvement in the Wnt signalling pathway, axon guidance, and stem cell pluripotency regulation. The DEGs in DIV 56 organoids are mainly involved in axon development, axonogenesis, and the development of neuronal projections. The KEGG pathway results are mainly involved in axon guidance, the MAPK signalling pathway, and the cell cycle. In the DIV 90 organoids, we found that the DEGs are primarily enriched in extracellular matrix and structural organisation, as well as various aspects of system development. KEGG pathway analysis found that the DEGs are mainly enriched in the JAK-STAT signalling pathway, PI3K-Akt signalling pathway, and neuroactive ligand−receptor interaction. In DIV 160 organoids, GO analysis of the biological processes revealed that the DEGs are mainly involved in chromosome segregation, nuclear division, and the mitotic cell cycle. KEGG pathway analysis showed that the DEGs are predominantly associated with the cell cycle, the HIF-1 signalling pathway, and the regulation of stem cell pluripotency. The full enrichment results and gene lists are provided in Supplementary Table S2.

### REST-targeted DEGs in different developmental stages of DS cerebral organoids

**Fig. 2:**
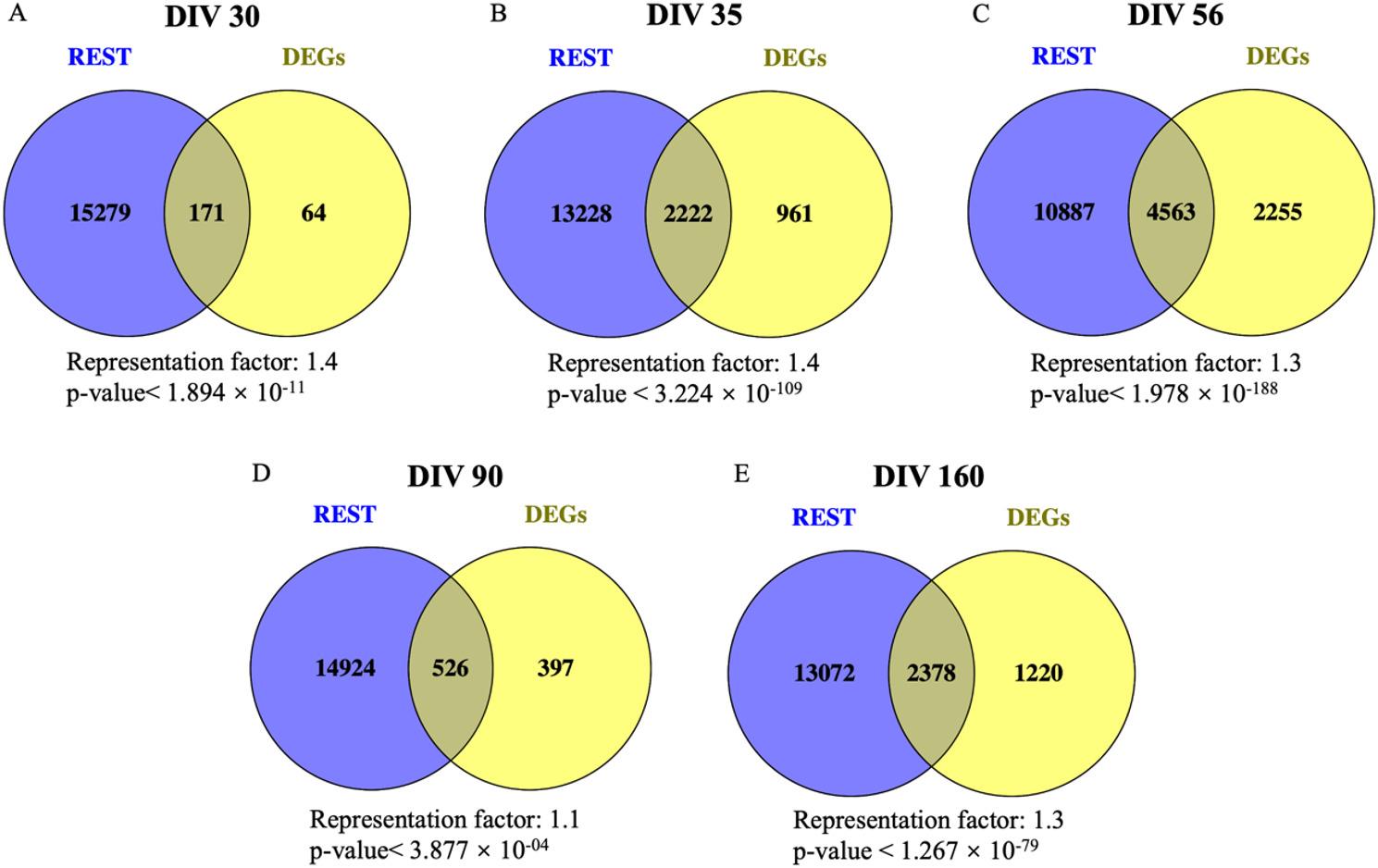
Depiction of the overlap between REST target genes and DEGs across the different time points of DS iPSC-derived organoids.**A** DIV 30 cerebral organoids; **B** DIV 35 cerebral organoids; **C** DIV 56 cerebral organoids; **D** DIV 90 cerebral organoids; **E** DIV 160 cerebral organoids. Blue circles represent REST target genes, yellow circles represent DEGs, and the overlaps represent REST target DEGs. Fisher’s test determined the statistical significance of this gene overlap, revealing all *p*-values < 0.01

This study comprehensively analysed overlapping genes between REST-target genes and DEGs in DS iPSC-derived organoids, and the results were visualised in Venn diagrams (Fig. 2). Our findings revealed a significant overlap, with 171 (72.77%) REST-targeted DEGs identified in DIV 30 organoids (representation factor, RF = 1.4 and *p* < 1.894 × 10^− 11^), 2,222 (69.81%) in DIV 35 organoids (RF = 1.4 and *p* < 3.224 × 10^− 109^), 4,563 (66.93%) in DIV 56 organoids (RF = 1.3 and *p* < 1.978 × 10^− 188^), 526 (84.43%) in DIV 90 organoids (RF = 1.1 and *p* < 3.877 × 10^− 04^), 2,378 (66.09%) in DIV 160 organoids (RF = 1.3 and *p* < 1.267 × 10^− 79^).

### REST as a pivotal regulator during DS iPSC-derived cerebral organoid development

**Fig. 3:**
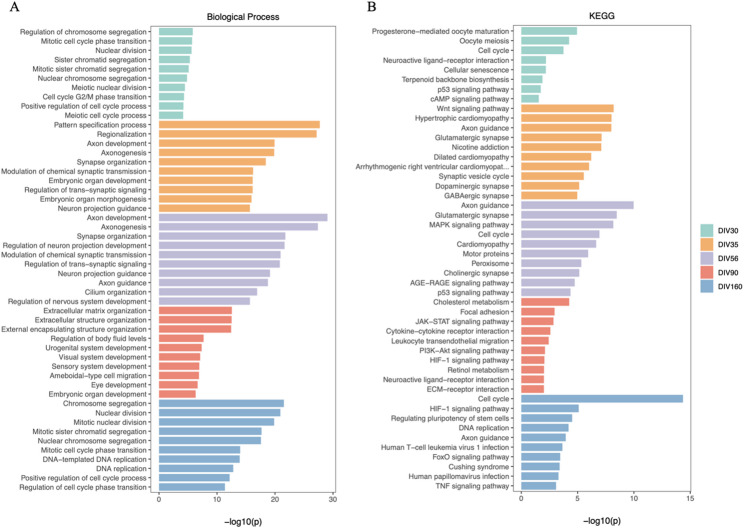
Analysis of REST target DEGs in DS-derived cerebral organoids at different time points.Different colours indicate different time points in DS cerebral organoids. The top 10 biological processes **A** and KEGG pathways **B** for each time point are shown

To further investigate the role of REST during DS iPSC-derived organoid development, GO and KEGG enrichment analyses were performed using REST-targeted DEGs identified at different time points (Fig. 3). Interestingly, REST-targeted DEGs in DIV 30 and DIV 160 DS iPSC-derived organoids involved similar biological functions and pathways, mainly enriched for chromosome segregation and cell cycle. In 35- to DIV 56 organoids, REST-targeted DEGs were associated with axon development, axon guidance, axonogenesis, neuron projection guidance, synapse organisation, dopaminergic synapse, GABAergic synapse, glutamatergic synapse, and the Wnt signalling pathway. At the same time, REST-targeted DEGs in DIV 90 organoids were notably enriched in focal adhesion, cytokine receptor interaction, PI3K-Akt, HIF-1, and JAK-STAT signalling pathways. The full enrichment results and gene lists are provided in Supplementary Table S2.

**Fig. 4:**
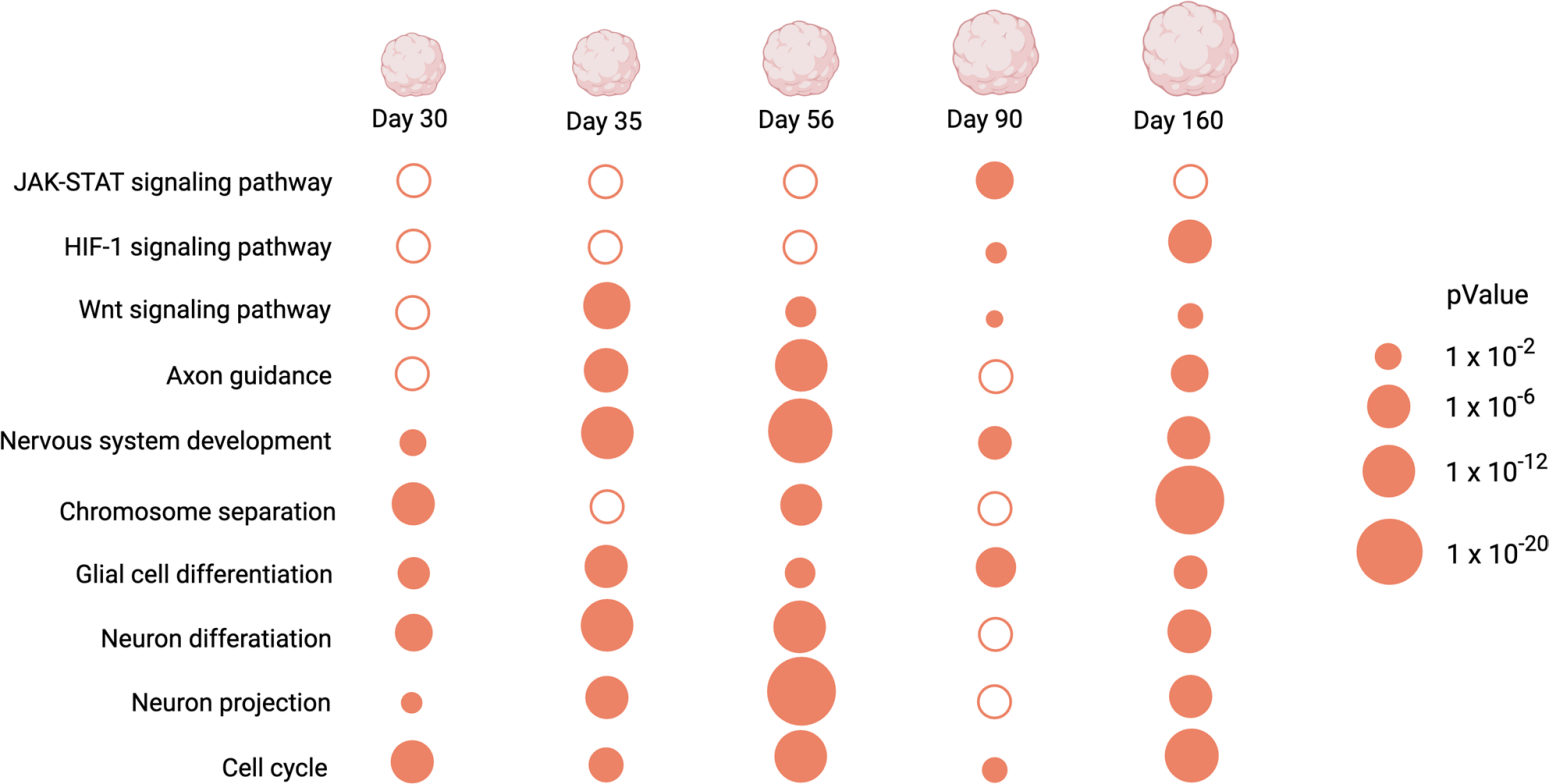
Summary of signalling pathways and biological processes associated with this Figure. Summary of selected signalling pathways and biological processes associated with REST-targeted DEGs in DS-derived cerebral organoids across developmental stages. The displayed terms were selected from the GO biological process and KEGG pathway enrichment results of REST-targeted DEGs at each developmental stage to summarize representative pathways relevant to neurodevelopment. The size of each circle represents the statistical significance of enrichment, based on the p-value from the enrichment analysis, with larger circles indicating lower p-values. Hollow circles indicate that the corresponding term was not significantly enriched at that developmental stage

The roles of REST-targeted DEGs in cerebral organoids at different time points are further summarised in Fig. 4. These terms were selected from the GO and KEGG enrichment analyses of REST-targeted DEGs at each stage to summarize representative pathways relevant to neurodevelopment. This analysis revealed that, in DIV 90 DS-derived cerebral organoids, the biological functions and signalling pathways involving REST-target DEGs were notably distinct from those at other time points. REST mainly targets the JAK-STAT signalling pathway and glial cell differentiation in DIV 90 DS-derived cerebral organoids. The full list of genes enriched in each pathway, along with the associated statistical results, is provided in Supplementary Table S3.

### GSEA revealed abnormal neurodevelopment in DS-derived DIV 90 cerebral organoids

**Fig. 5:**
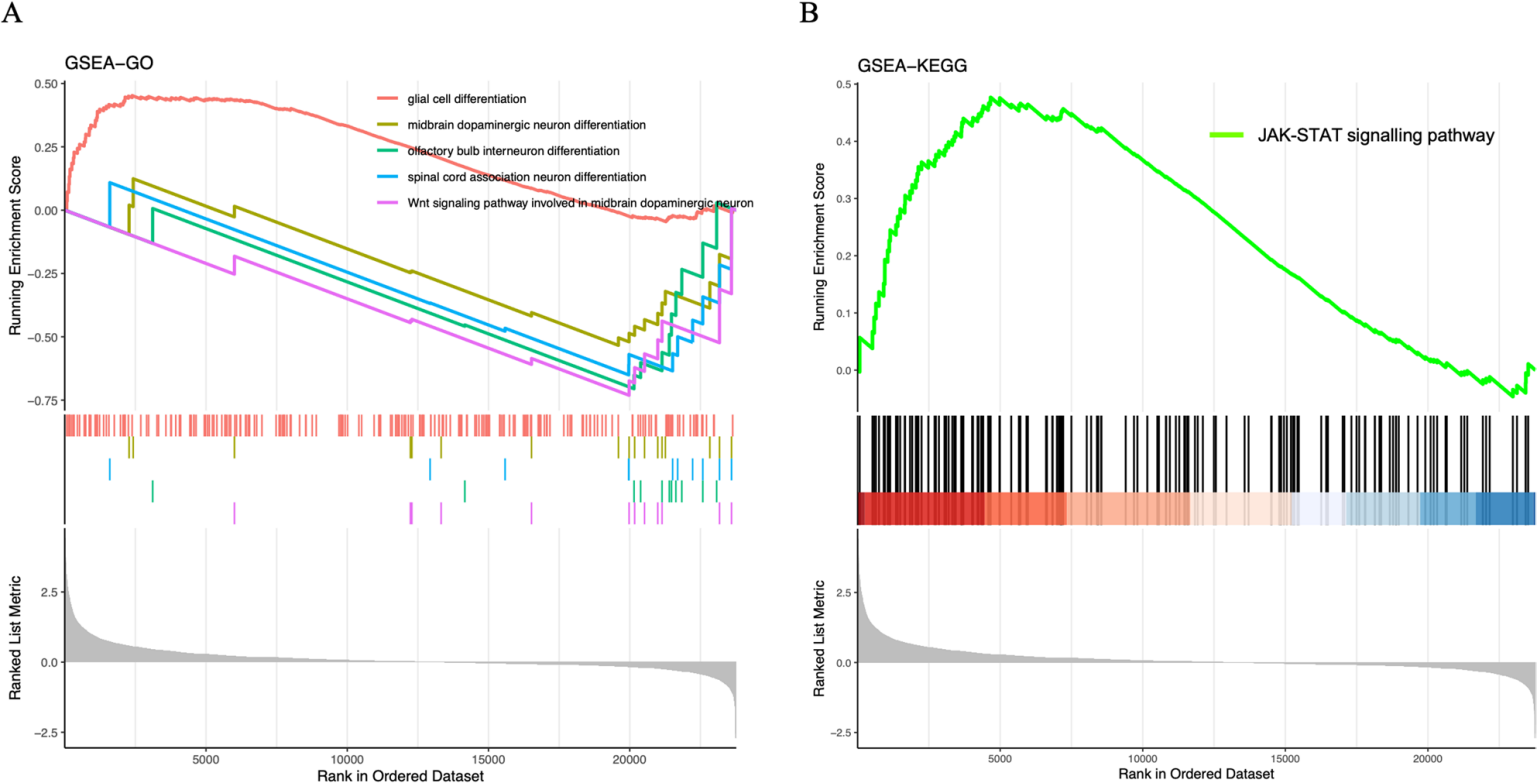
GSEA analysis for DIV 90 DS cerebral organoids.**A** GSEA-GO and **B** GSEA-KEGG analysis for the DEGs in DS-derived cerebral organoids at different time points. All results shown are |NES| >1 and adjusted *p* < 0.05. The graphical representation of the GSEA analysis results is divided into three parts. The top section displays the enrichment score (ES) curves, where different coloured curves represent the ES of gene sets associated with various biological processes and signalling pathways within the ranked gene list (expression matrix). A positive ES indicates that the gene set is enriched at the top of the list, indicating upregulated pathways. In contrast, a negative ES indicates enrichment at the bottom, indicating downregulated pathways. The middle section contains coloured bars, each line representing a gene from the corresponding gene set of biological processes and signalling pathways, indicating its position in the ranked gene list. The bottom section shows the distribution of gene expression levels within the ranked gene list, providing a visual representation of gene expression across the dataset

To further understand the biological characteristics of DIV 90 cerebral organoids, GSEA analyses were performed. GSEA-GO analysis revealed defects in neuronal differentiation and increased glial cell differentiation in DS-derived DIV 90 organoids. Several gene sets associated with neuronal differentiation programs were significantly downregulated in DS-derived DIV 90 organoids, including midbrain dopaminergic neuron differentiation, olfactory bulb interneuron differentiation, spinal cord association neuron differentiation, and the Wnt signalling pathway involved in midbrain dopaminergic neuron differentiation (Fig. 5). Conversely, several gene sets associated with glial cell differentiation programs were upregulated. These GO-related gene sets were significantly enriched for NES < -1, and adjusted p-value < 0.05. Further GSEA-KEGG analysis indicated that the JAK-STAT signalling pathway was upregulated in DS-derived DIV 90 organoids with NES > 1, p-value < 0.05, and adjusted p-value < 0.05 (Fig. 5B). The JAK-STAT signalling pathway is widely recognised as essential for regulating the proliferation of neural progenitor cells and the differentiation of astrocytes.

### WGCNA identified critical module in DS-derived DIV 90 cerebral organoids

**Fig. 6:**
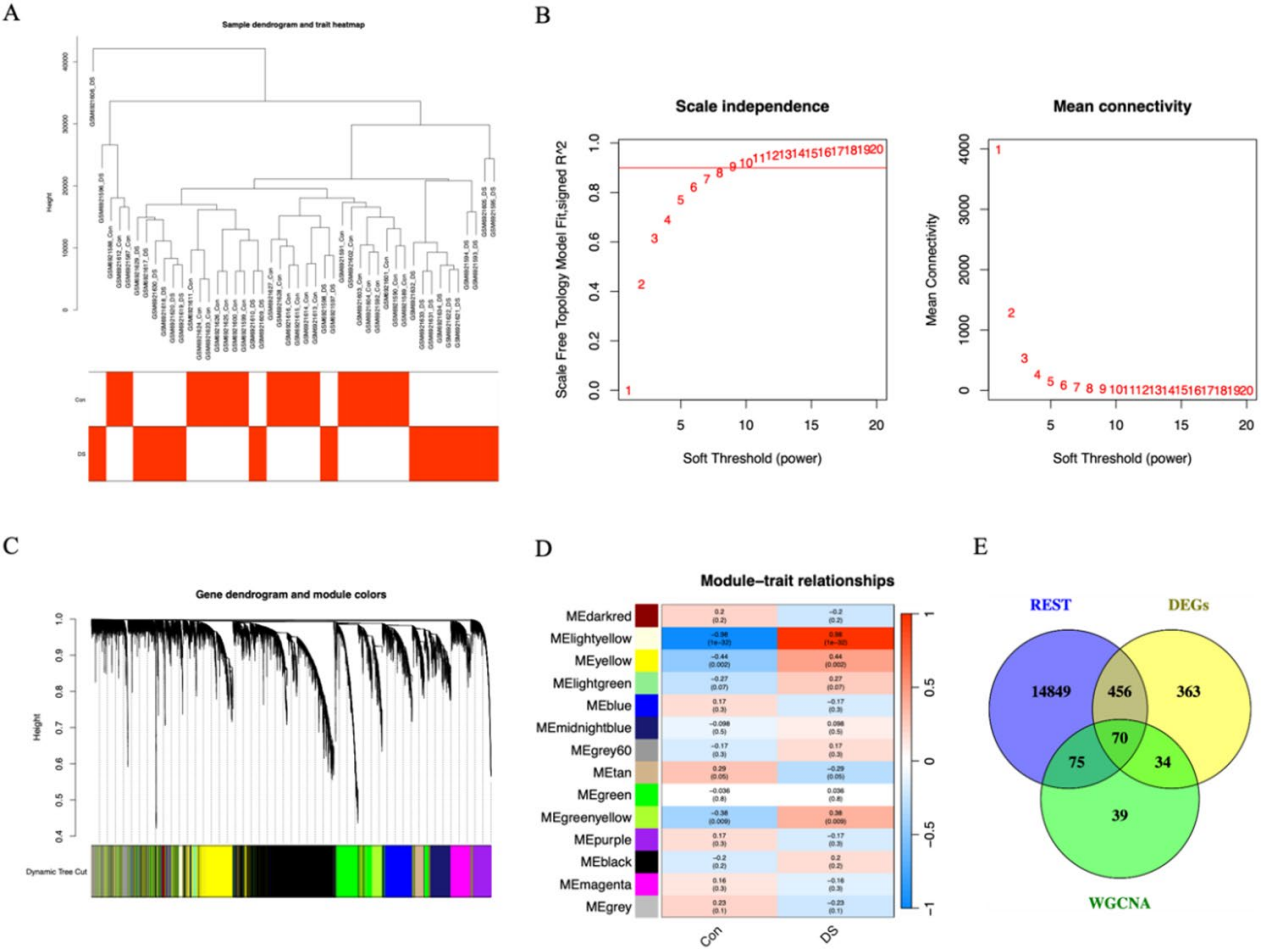
WGCNA unveiled gene co-expression networks and module-trait relationships. **A** Sample clustering was used to identify outliers, and a feature heatmap was used to visualise sample characteristics. **B** The network topology was analysed across different soft-threshold powers. The graph on the left shows the relationship between the scale-free fit index (y-axis) and the soft-threshold powers (x-axis). The graph on the right shows the relationship between mean connectivity (degree, y-axis) and soft-threshold powers (x-axis). A power value (β) of 9 was set for further analysis. **C** Clustering dendrograms of all genes based on topological overlap and assigned module colours were constructed, resulting in 14 distinct co-expression modules, each represented by a different colour. The image displays the relationship between the gene dendrogram at the top and the gene modules at the bottom. **D** The analysis examined the relationships between modules and traits. The table displays correlations and P-values with different traits for each module eigengene. The brown module was identified as the most significantly associated with DS. **E** A Venn diagram illustrates the overlap between REST-targeted DEGs and the genes in the light-yellow module

DS-related critical genes in iPSC-derived DIV 90 organoids were identified by constructing scale-free co-expression networks using the R package “WGCNA”. The GSE222365 dataset was selected for clustering evaluation. Hierarchical clustering of samples was performed on expression values using average linkage and Pearson correlation to identify sample outliers. The resulting samples were then analysed by clustering and phenotypic heat map (Fig. 6A). A power value of β = 9 was selected as the soft threshold to construct a scale-free network for the gene expression network (Fig. 6B). A hierarchical clustering tree was constructed from the neighbour-joining matrix and the TOM matrix of gene pairs (Fig. 6C). Fourteen co-expression modules were identified from the expression profiles of DS brain organoids, with distinct colours representing each module. Pearson correlation analysis was conducted for the genes within each module across various groups (Fig. 6D). Correlation analysis of module features revealed that the MElightyellow module, comprising 218 genes, exhibited the strongest association with DS, achieving a correlation coefficient of 0.98. REST-targeted DEGs were compared with MElightyellow module genes to further screen hub genes in REST-targeted DEGs, and 70 overlapping genes were obtained for subsequent analysis (Fig. 6E).

### Machine learning for identifying critical genes

**Fig. 7:**
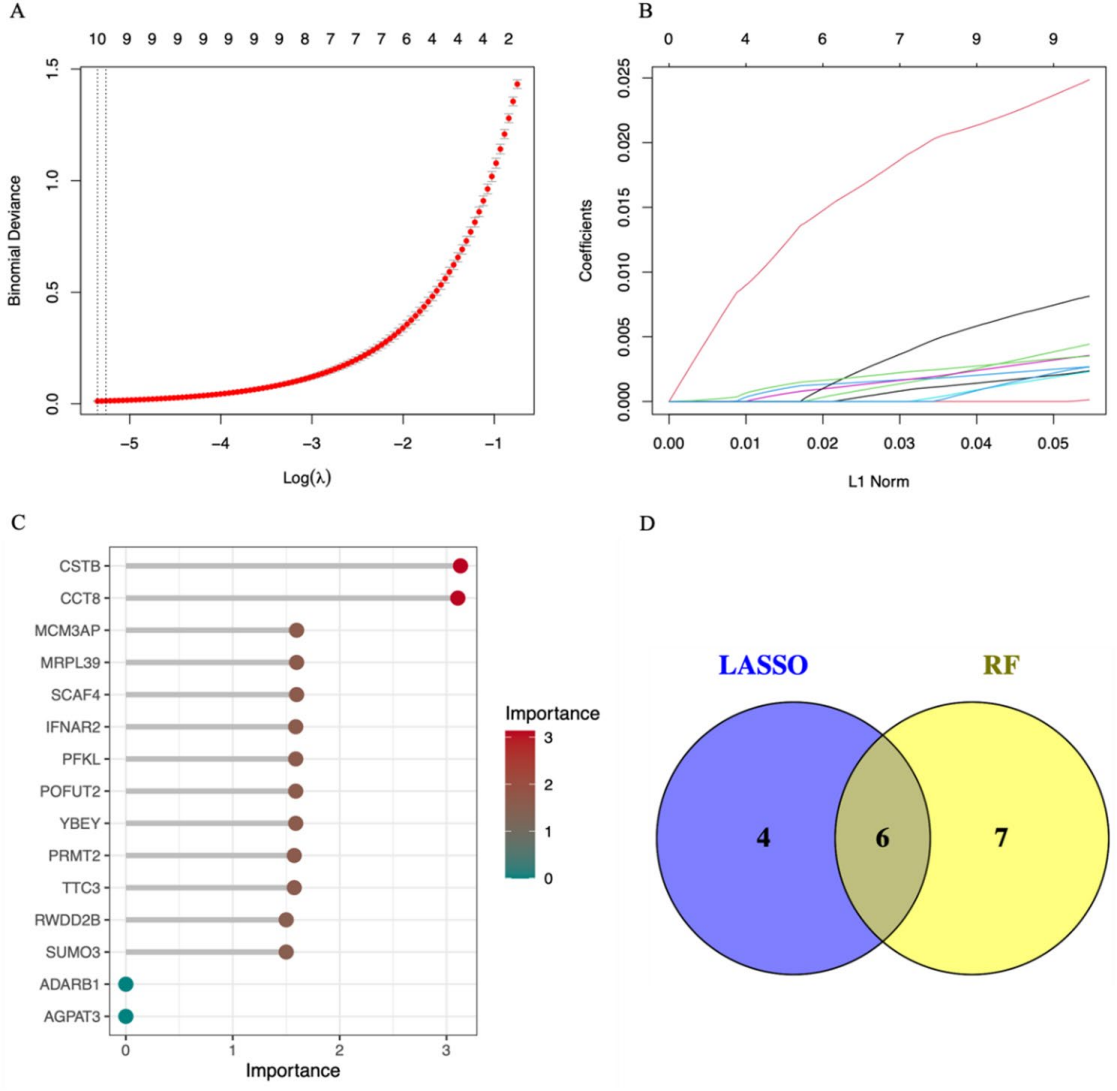
Screening hub genes from REST target DEGs in DIV 90 DS iPSC-derived cerebral organoids. **A**–**B** The LASSO algorithm screened 10 hub genes. **C** The RF algorithm screened 13 diagnostic markers. **D **Venn diagram of 6 genes, including *CSTB*,* MCM3AP*,* PFKL*,* POFUT2*,* PRMT2*, and *RWDD2B*, intersected by LASSO and RF algorithms

To further identify hub genes associated with DS development, the 70 REST-targeted DEGs that overlapped with the WGCNA core module in DIV 90 organoids were selected for hub gene screening. We used the LASSO logistic regression algorithm to identify 10 significant feature genes related to DS from the overlapping genes (Fig. 7A–B). The RF algorithm identified 13 significant feature genes (Fig. 7C). A total of 6 overlapping genes (*CSTB*, *MCM3AP*, *PFKL*, *POFUT2*, *PRMT2*, and *RWDD2B*) were identified by comparing the LASSO and RF analysis results (Fig. 7D).

### In silico and laboratory validation of REST-targeted hub DEGs

**Fig. 8:**
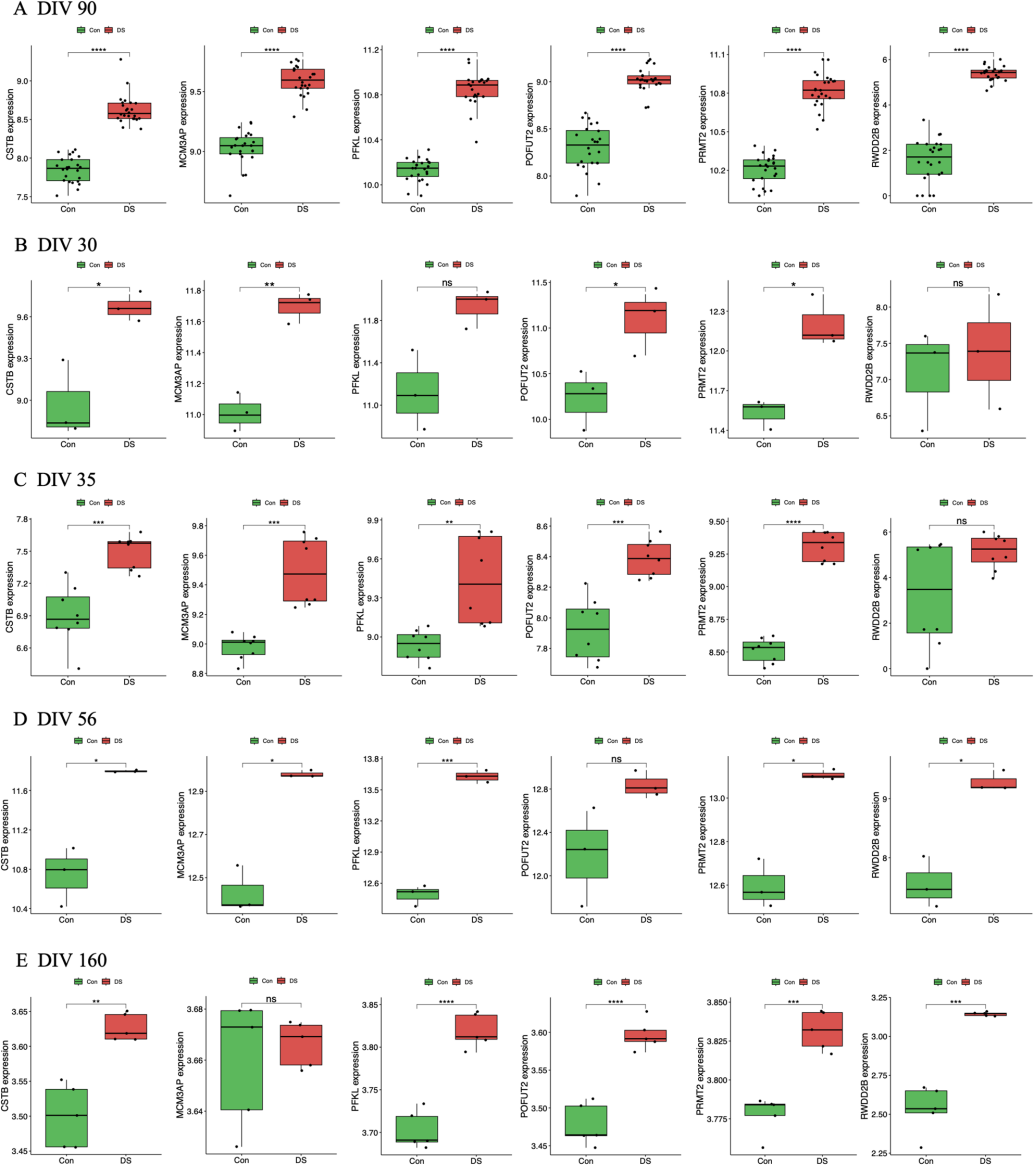
In silico analysis for REST-targeted hub DEGs identified at DIV 90 DS-derived cerebral organoids.**A–E** Statistical comparison of the expression levels of six hub genes (*CSTB*, *MCM3AP*, *PFKL*, *POFUT2*, *PRMT2*, and RWDD2B) between DS-derived and control cerebral organoids at different developmental stages (DIV 90, DIV 30, DIV 35, DIV 56, and DIV 160). Differential expression was evaluated using a two-tailed t-test

Because the hub genes were identified from the DIV 90 transcriptomic network, their expression levels were first evaluated in the DIV 90 organoid dataset. Statistical analysis showed that all six hub genes (*CSTB*, *MCM3AP*, *PFKL*, *POFUT2*, *PRMT2*, and *RWDD2B*) were significantly upregulated in DS-derived cerebral organoids compared with controls at DIV 90 (Fig. 8A). To further examine whether these genes exhibited similar transcriptional patterns during organoid development, their expression levels were subsequently analysed in additional datasets corresponding to DIV 30, DIV 35, DIV 56, and DIV 160. As shown in Fig. 8B–E, most hub genes exhibited higher expression in DS-derived organoids than in controls across these developmental stages. However, not all genes reached statistical significance at every time point. Together, these findings indicate that the identified REST-targeted hub genes tend to show elevated expression in DS cerebral organoids during development, with the DIV 90 stage representing the key developmental window in which all hub genes exhibited significant dysregulation.

#### Laboratory validation of REST-targeted hub DEGs

**Fig. 9:**
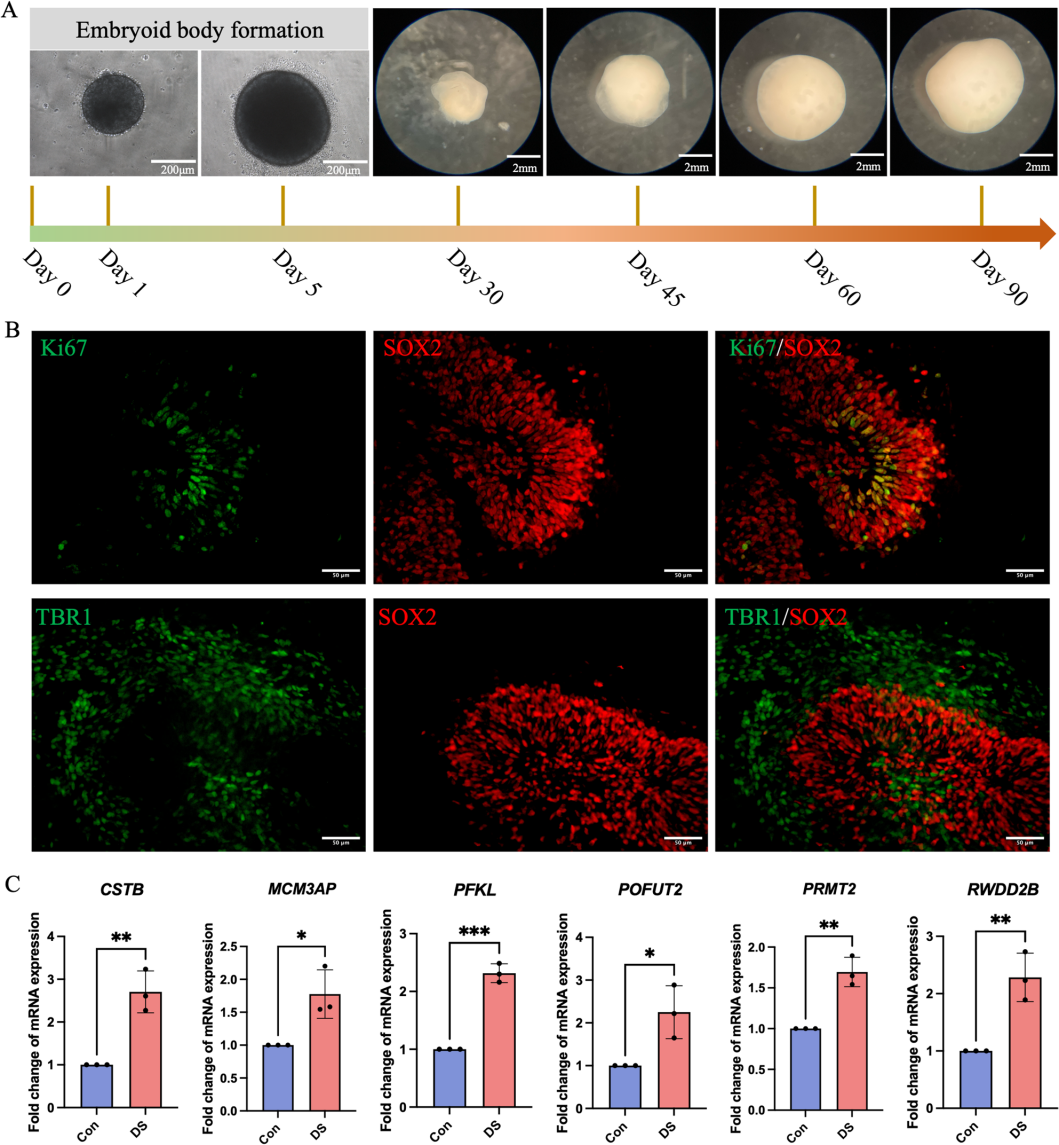
Culture of cerebral organoids and validation of REST-targeted hub gene in DIV 90 cerebral organoids. Three pairs of cell lines (C1 vs. DS1, C2 vs. DS2, and C5 vs. DS4) were used to perform qPCR analysis. **A** Culture timeline and morphology of DIV 90 cerebral organoids. **B** Immunochemical staining of a DIV45 cerebral organoid showed positive expression of Ki67, SOX2, and TBR1. **C** *CSTB*, *MCM3AP*, *PFKL*, *POFUT2*, *PRMT2*, and RWDD2B mRNA levels were detected by RT-qPCR. The mean ± SD is from experiments of three independent cell lines. *P < 0.05, **P < 0.01, ***P < 0.001. Con, Control; DS, Down syndrome

To validate the predicted REST-targeted hub genes, cerebral organoids derived from three control and three DS hiPSC lines were cultured for further analysis. The first 5 days of culture are the period of EB formation (EB), and EBs on the 5th day should have a diameter greater than 300 μm and rounded edges, which is conducive to neural induction and growth of cerebral organoids in the later stage. The cerebral organoids grew in size as they were incubated for longer periods in subsequent cultures (Fig. 9A). On DIV 45, immunocytochemistry was performed to assess the growth and development of cerebral organoids. Positive results for neural rosette formation and inside-out cortical layering, as indicated by the cell proliferation marker Ki67, the neural progenitor marker SOX2, and the neuronal migration marker TBR1, indicated that the organoids were well developed (Fig. 9B). All organoids were harvested for RNA extraction and cDNA synthesis at 90 days old. To verify REST-targeted hub gene expression, RT-qPCR was performed to assess mRNA levels of *CSTB*,* MCM3AP*,* PFKL*,* POFUT2*,* PRMT2* and *RWDD2B*. Our study showed that mRNA levels of *CSTB*,* MCM3AP*,* PFKL*,* POFUT2*,* PRMT2*, and *RWDD2B* were significantly upregulated by 1.4-3.2-fold in DS-derived DIV 90 cerebral organoids compared to the control group (Fig. 9C).

### Altered neuronal and glial developmental marker expression in DIV 90 DS cerebral organoids

**Fig. 10:**
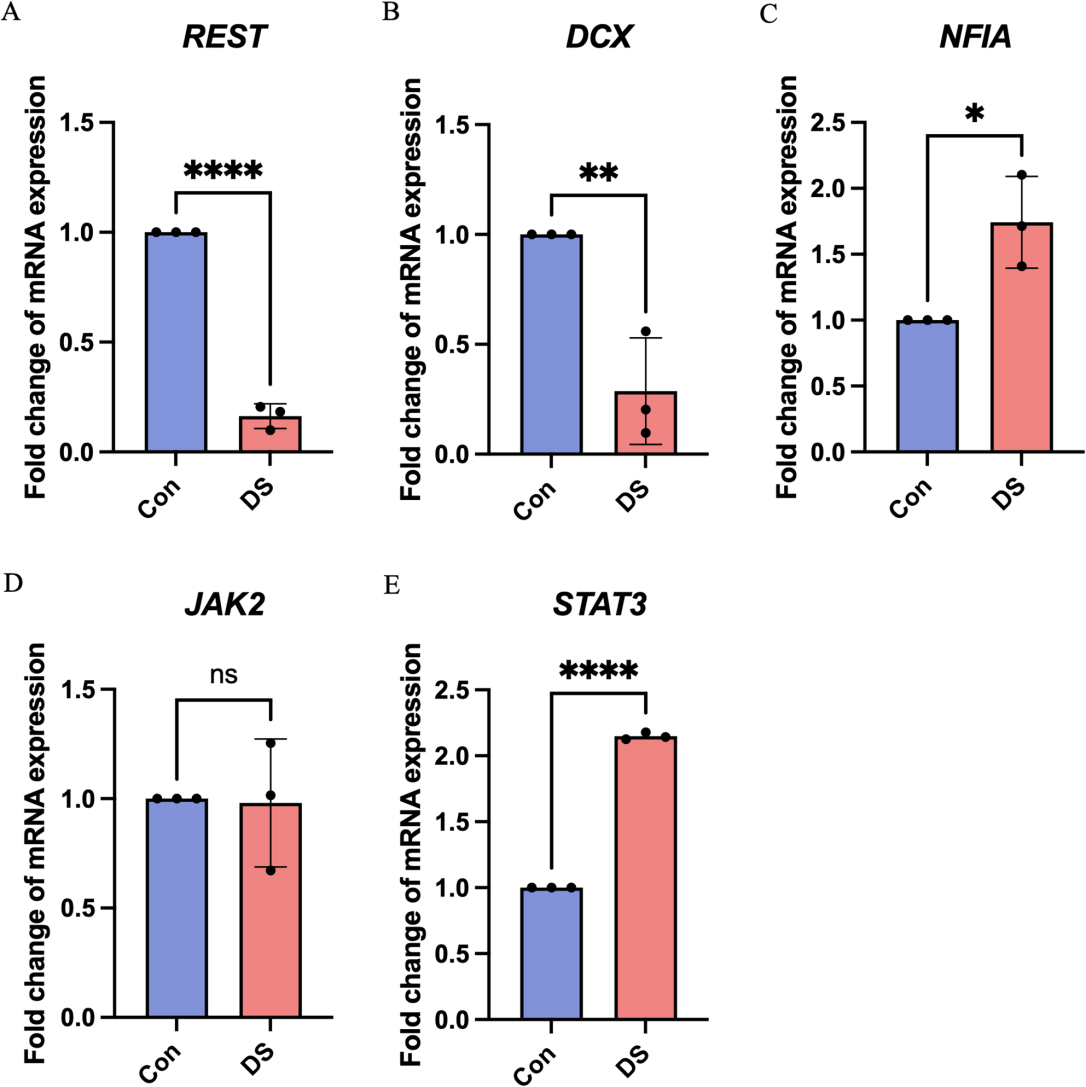
Detection of REST and neural differentiation-related genes in DIV 90 DS iPSC-derived cerebral organoids. Three pairs of cell lines (C1 vs. DS1, C2 vs. DS2, and C5 vs. DS4) were used to perform qPCR analysis. All measurements were normalised by dividing DS values by the corresponding controls. **A**–**E** The expression levels of *REST*, *DCX*, *NFIA*, *JAK2*, and *STAT3* mRNA were detected by qPCR in the DS and control groups, respectively

**Fig. 11:**
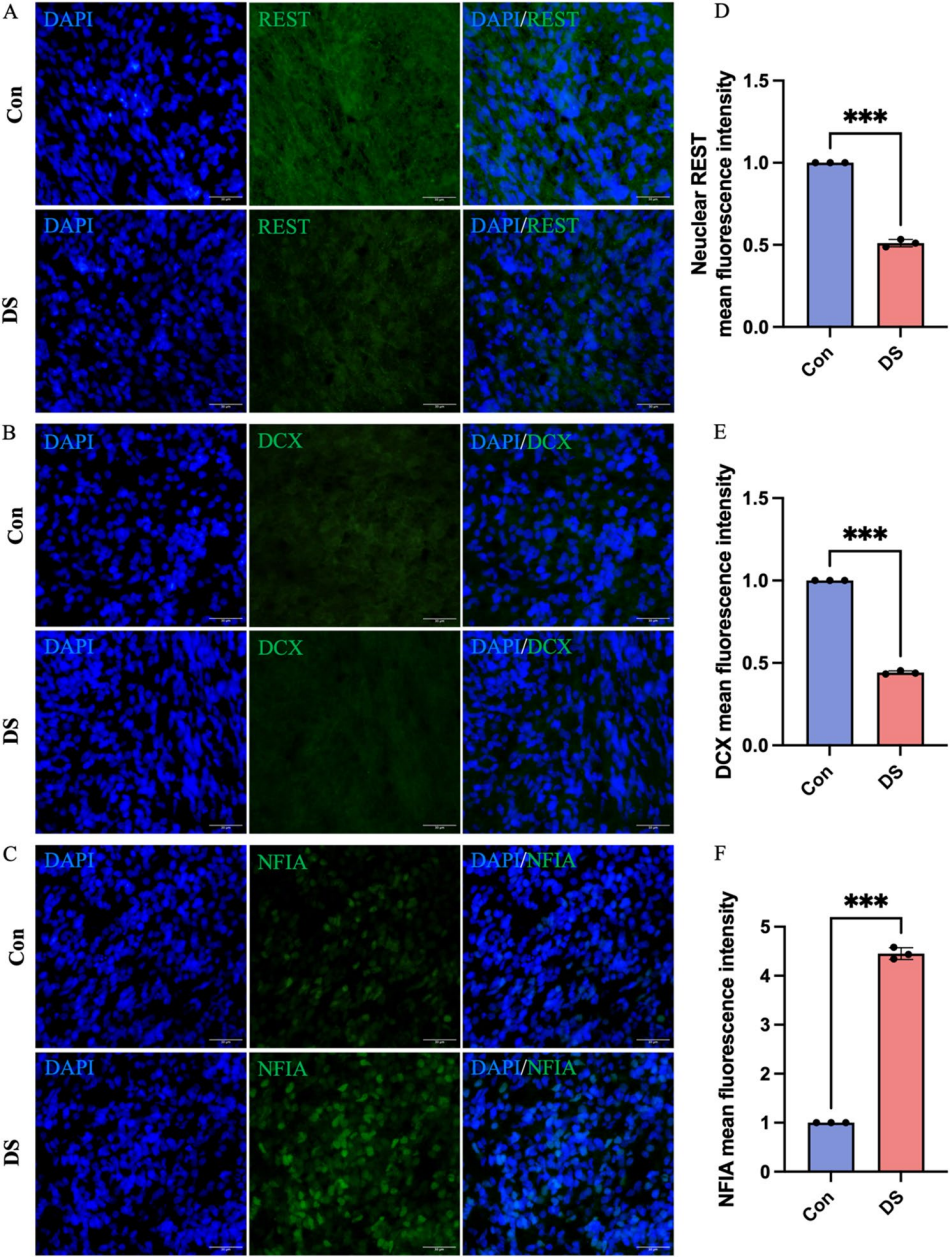
Immunofluorescence staining of REST, DCX, and NFIA in DIV 90 cerebral organoids. Three pairs of cell lines (C1 vs. DS1, C2 vs. DS2, and C5 vs. DS4) were used to perform immunochemical staining analysis. Representative immunofluorescence images showing the expression of REST, DCX, and NFIA in control and DS hiPSC-derived cerebral organoids at DIV 90. Nuclei were stained with DAPI (blue), and the indicated proteins were detected using specific antibodies (green). Merged images show the overlay of DAPI and the corresponding marker. **A–C** REST immunostaining was detected in both the nucleus and cytoplasm, consistent with previous reports that REST can undergo nuclear–cytoplasmic shuttling in neural cells. Compared with control organoids, DS-derived organoids exhibited reduced REST and DCX staining and increased NFIA staining. **D**–**F** Quantification of fluorescence intensity is shown on the right. Data are presented as mean ± SD. Statistical significance was determined using a two-tailed Student’s t-test (*p* < 0.05). Scale bars = 30 μm. *P < 0.05, **P < 0.01, *** *P* < 0.001, **** *P* < 0.0001. Con, Control; DS, Down syndrome

To verify the role of REST in cerebral organoids, qPCR and immunostaining were performed to detect REST mRNA and protein levels, respectively. The results showed that the expression levels of REST mRNA and nuclear REST protein in DIV 90 DS iPSC-derived cerebral organoids were significantly lower than those in the control group (Figs. 10A and 11A and B). The mRNA expression level of REST in DS iPSC-derived cerebral organoids was only 16.39% of the level in the control group. To further examine neurodevelopmental changes associated with REST downregulation, the neuroblast marker DCX and the glioblast marker NFIA were evaluated. The results revealed that DCX mRNA levels were downregulated by 3.8-fold and protein levels by 2.3-fold in DIV 90 DS iPSC-derived cerebral organoids compared with controls (Figs. 10B and 11C and D). At the same time, the expression levels of NFIA mRNA and protein were upregulated 1.7- and 4.5-fold, respectively, in DIV 90 DS iPSC-derived cerebral organoids compared to controls (Figs. 10C and 11E and F). These findings indicate altered expression of neuronal and glial developmental markers in DIV 90 DS cerebral organoids and are consistent with transcriptional changes in neurodevelopmental programs at this stage. To explore whether the JAK–STAT signalling pathway might be involved in this process, the mRNA expression of JAK2 and STAT3 was assessed. Although this study found no statistically significant difference in the expression of JAK2 mRNA between DIV 90 DS iPSC-derived cerebral organoids and controls (Fig. 10D), the expression of STAT3 mRNA was significantly upregulated by 2.14-fold compared to controls (Fig. 10E). These findings suggest that JAK–STAT signalling may be associated with the transcriptional changes observed in DIV 90 DS cerebral organoids.

## Discussion

In this study, we analysed bulk transcriptomic data from DS and control hiPSC-derived cerebral organoids across five developmental stages (DIV30, DIV35, DIV56, DIV90, and DIV160) and identified dynamic transcriptional alterations across organoid maturation. Our enrichment analyses indicated that DS-associated transcriptional abnormalities were not static, but shifted across developmental time, with early-stage signatures involving cell-cycle-related processes and neuronal developmental pathways, and a later-stage prominence of glial-associated programs around DIV90.

A key point in interpreting these findings is the nature of the cerebral organoid model itself. The protocol used here follows a self-organizing cerebral organoid paradigm, which is well known to generate heterogeneous neuroepithelial domains and multiple region-like identities rather than a single uniformly specified brain region [27]. Cerebral organoids generated using unguided protocols were originally shown to develop various discrete, though interdependent, brain-region-like domains, and later work further demonstrated broad neural cell-type diversity and asynchronous maturation within individual organoids [27, 28]. Therefore, the diverse lineage-related and cell-type-associated signals observed in our dataset are consistent with the established biological properties of cerebral organoids and should be interpreted in the context of multi-domain organization and developmental heterogeneity rather than as evidence of a single homogeneous lineage trajectory [27, 28].

Within this framework, the recurrent enrichment of cell-cycle-related pathways in DS samples is particularly notable. In our dataset, cell-cycle-associated processes were enriched at DIV30 and re-emerged at DIV160. At the same time, REST-targeted DEGs at these stages likewise showed enrichment for chromosome segregation and mitotic cell-cycle programs. Rather than indicating only increased proliferation, this pattern may reflect delayed differentiation, impaired cell-cycle exit, prolonged retention of progenitor-like states, or asynchronous maturation in DS organoids relative to controls. This interpretation is supported by prior work showing that trisomy 21 neural progenitors display altered developmental progression and abnormal proliferative control, including impaired neurogenesis in DS cerebral organoids and a biphasic cell-cycle defect linked to defective neuronal output [2, 29]. Because long-term cerebral organoid cultures also retain proliferative progenitor populations and exhibit marked developmental heterogeneity, later-stage cell-cycle enrichment in DS organoids may reflect a transcriptomic signature of delayed developmental progression, superimposed on the intrinsic heterogeneity of the organoid system [28].

The emphasis on DIV90 in the present study should also be interpreted from both biological and analytical perspectives. Biologically, DIV90 corresponds to a developmental window during which gliogenic programs become more apparent in many cerebral or cortical organoid systems, making this stage especially relevant for evaluating the observed neurogenic-to-gliogenic shift in DS organoids [28]. Consistent with this, our GSEA results at DIV90 indicated downregulation of neuronal differentiation-related gene sets, along with upregulation of glial differentiation-related pathways, and our experimental validation showed reduced DCX expression, with increased NFIA and STAT3 expression, in DS organoids at this stage. At the same time, we acknowledge that DIV90 was also the most suitable time point for network analysis from a statistical standpoint, because the GSE222365 dataset included the largest number of samples at DIV90 (24 DS and 24 controls), which likely improved the robustness and stability of WGCNA compared with other available developmental stages. Accordingly, the prominence of DIV90 in our study likely reflects both a biologically informative transition toward gliogenic differentiation and the stronger analytical power available at this time point.

Taken together, these findings support a unified interpretation of our data. DS cerebral organoids appear to exhibit persistent dysregulation of developmental timing, reflected by recurrent cell-cycle signatures, altered neuronal differentiation, and a pronounced shift toward gliogenic programs around DIV90. In a self-organizing, multi-domain organoid system, these abnormalities are likely expressed through both delayed lineage progression and altered timing of the neurogenic-to-gliogenic transition, rather than through a single isolated pathogenic event. This interpretation is consistent with the broader view that impaired neurogenesis in DS is tightly linked to abnormal developmental timing and progenitor-state regulation [2, 29].

To further investigate transcriptional regulators underlying these developmental alterations, we identified six REST-targeted hub genes, namely *CSTB*, *MCM3AP*, *PFKL*, *POFUT2*, *PRMT2*, and *RWDD2B*, through integrated WGCNA and machine-learning analyses. These genes were identified from the DIV90 transcriptional network and may represent candidate regulatory nodes associated with REST-related transcriptional dysregulation during DS organoid development. Notably, RWDD2B is reported here for the first time in the context of DS, highlighting the need for further investigation into its neurodevelopmental role. Overexpression of CSTB has been reported to enhance neural progenitor proliferation while perturbing cell-cycle progression and neuronal commitment [30]. In contrast, PFKL dysregulation has been linked to altered glucose metabolism and neurodevelopmental defects in DS-related contexts [31]. In addition, POFUT2-related pathways have been implicated in cell-fate regulation and neuronal morphogenesis in developmental systems, while arginine methyltransferase activity has been associated with STAT3-related astroglia programs [32]. Together, these findings suggest that aberrant expression of REST-targeted hub genes may contribute synergistically to the imbalance between neuronal production and glial expansion observed in DS, providing candidate molecular entry points for future mechanistic studies.

REST is a transcription factor that controls pluripotency and cell fate and is widely recognised as a key regulator of embryonic and neural development [10, 15]. By repressing the expression of target genes, REST regulates neurogenesis, maturation, and neuroprotection, and its activity depends on a broader co-repressor network that includes CoREST and chromatin-modifying machinery [33, 34]. Our analyses across multiple developmental stages suggest that REST-related transcriptional networks may be broadly dysregulated during DS cerebral organoid development. Rather than regulating a single pathway, REST appears to operate within a complex regulatory network involving numerous target genes, the REST corepressor, and other transcription factors [35]. In this context, the widespread enrichment of REST-targeted DEGs across developmental stages supports the view that REST dysregulation may represent a broader organizing principle underlying lineage imbalance in DS organoids.

Based on the transcriptional alterations observed at DIV90, we further examined REST expression and neurodevelopmental marker expression in DS cerebral organoids. We observed significant downregulation of REST expression, reduced expression of the neuroblast marker DCX, and increased expression of the glioblast marker NFIA in DS organoids compared with controls. These findings are consistent with the RNA-sequencing results and support the presence of altered neuronal and glial developmental programs at this stage. Previous studies have shown that reduced REST activity can de-repress neuronal genes and transiently promote premature neuronal differentiation [36, 37]; however, sustained or inappropriate REST loss may impair maintenance of the neural progenitor pool, ultimately reducing effective neurogenesis and disturbing lineage progression [13, 15]. Thus, our findings highlight the importance of appropriate REST dosage and timing during neural development, particularly during the transition from neurogenic to gliogenic competence.

In addition to its role in neuronal gene regulation, REST also influences the epigenetic landscape of glial cells [14, 38]. Although previous studies have mainly focused on REST in neurogenesis [39, 40], its role in gliogenesis is increasingly recognised. In our DS organoids, increased NFIA expression suggests that REST loss may be associated with enhanced gliogenic bias. However, the effects of REST on astroglia differentiation are likely context dependent. Abrajano et al. showed that REST and CoREST are differentially deployed during glial subtype specification and oligodendrocyte lineage maturation, supporting the idea that REST loss in DS cerebral organoids could derepress glial-associated transcriptional programs and contribute to increased astrogenesis [14]. Our results are therefore consistent with a model in which REST deficiency contributes not only to impaired neuronal developmental progression but also to a relative bias toward gliogenic programs.

Transcriptomic analysis also suggested enrichment of JAK–STAT-related pathways in DIV90 DS cerebral organoids. Consistent with this observation, an analysis showed increased STAT3 expression in DS organoids compared with controls, whereas JAK2 expression did not differ significantly between groups. These findings raise the possibility that STAT3 activation in DS organoids may involve non-canonical upstream regulation rather than a simple JAK2-driven mechanism, although this will require further mechanistic investigation [41, 42]. More broadly, STAT3 is a central mediator of astrogenic signalling and interacts with pathways such as BMP and Notch to promote astrocytic differentiation. At the same time, DYRK1A overexpression in a DS mouse model has been shown to enhance STAT activity and astrogliogenesis [4]. The observed increase in STAT3 expression, together with elevated NFIA and glial differentiation signatures, therefore supports the idea that REST dysregulation in DS organoids is associated with a shift toward gliogenic programs at DIV90. Nevertheless, further studies are required to define the precise mechanistic relationship between REST deficiency and JAK–STAT pathway activation in this context.

## Conclusion

This study provides an integrated view of neurodevelopmental dysregulation in DS across distinct developmental stages using hiPSC-derived cerebral organoids. Our findings reveal altered neurodevelopmental programs characterised by reduced neuronal differentiation signals and increased glial-associated pathways, particularly at DIV 90. Central to these changes is the dysregulation of REST and its downstream transcriptional network. These results highlight REST as an important regulator of transcriptional programs during DS brain development and suggest that the DIV 90 cerebral organoid stage may represent a critical window for investigating disease mechanisms and potential therapeutic strategies. Future studies will be required to determine the precise molecular pathways through which REST dysregulation influences neurodevelopmental trajectories and whether modulation of REST activity may help restore neurodevelopmental balance in DS.

## Supplementary Information

Below is the link to the electronic supplementary material.

Supplementary Material 1

## Data Availability

The original contributions presented in the study are included in the article/Supplementary material; further inquiries can be directed to the corresponding author. All data sets used in this study are publicly available on the Gene Expression Omnibus (GEO) and the Sequence Read Archive (SRA). The accession numbers are GSE124513, GSE208440, GSE222365, and PRJNA721827 (SRR14244005-SRR14244010).

## Abbreviations

DEGs: Differentially Expressed Genes
DS: Down syndrome
GEO: Gene expression omnibus database
GO: Gene ontology
GS: Gene significance
GSEA: Gene set enrichment analysis
hiPSC: human induced pluripotent stem cells
KEGG: Kyoto encyclopedia of genes and genomes
LASSO: Least absolute shrinkage and selection operator
MM: Module membership
NGS: Next-Generation Sequencing
RF: Random Forest^1^; Representation Factor^2^
WGCNA: Weighted gene co-expression network analysis
