# Supplementary material for: REST deficiency and neurogenic-to-gliogenic shift in down syndrome human cerebral organoids
Source: Mol Brain. 2026 May 16;19:55. doi: 10.1186/s13041-026-01313-2 (PMC13348641; doi:10.1186/s13041-026-01313-2)
Supplement: Supplementary file 1 — Supplementary Material 1 [file 13041_2026_1313_MOESM1_ESM.zip › Supplementary Information.pdf]

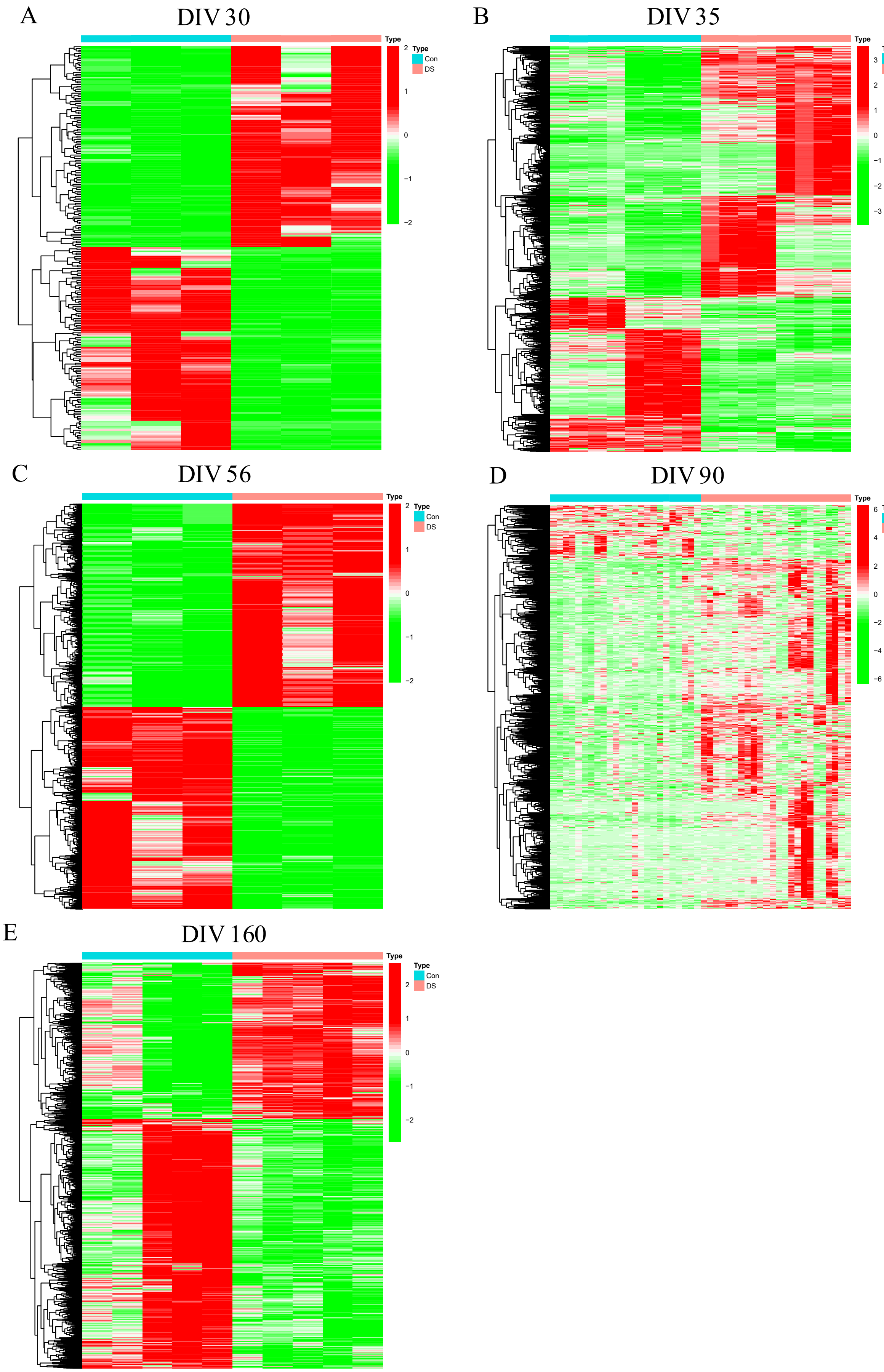

Figure S1. Heatmap visualization of differentially expressed genes (DEGs) in DS iPSC-derived cerebral organoids across developmental stages. (A) Heatmap of DEGs identified at DIV 30 (PRJNA721827 dataset). (B) Heatmap of DEGs identified at DIV 35 (GSE124513 dataset). (C) Heatmap of DEGs identified at DIV 56 (GSE208440 dataset). (D) Heatmap of DEGs identified at DIV 90 (GSE222365 dataset). (E) Heatmap of DEGs identified at DIV 160 (GSE222365 dataset). Red indicates higher expression and green indicates lower expression.

Supplementary Table S1. Differentially expressed genes identified in DS cerebral organoids across developmental stages.

This table contains five sheets corresponding to DIV 30, DIV 35, DIV 56, DIV 90, and DIV 160. Each sheet lists all differentially expressed genes identified at the respective developmental stage, including gene symbol, log2 fold change, and adjusted p-value.

Supplementary Table S2. Full GO and KEGG enrichment results and associated gene lists for the DEGs shown in Figure 1.

This table provides the enrichment results for the GO biological processes and KEGG pathways identified from DEGs and REST target DGEs at different developmental stages of DS-derived cerebral organoids in Figure 1 and Figure 3. The associated genes and statistical enrichment results, including p-values and adjusted p-values, are listed.

Supplementary Table S3. Gene lists and statistical enrichment results for the GO and KEGG pathways summarized in Figure 4.

The table includes genes enriched in enrichment results for the GO and KEGG pathways identified from REST target DGEs at different developmental stages of DS-derived cerebral organoids shown in Figure 4.
